# Stability Assessment in Aqueous and Organic Solvents of Metal–Organic Framework PCN 333 Nanoparticles through a Combination of Physicochemical Characterization and Computational Simulations

**DOI:** 10.1021/acs.langmuir.4c01684

**Published:** 2024-10-12

**Authors:** Xiaoli Liu, Andres Ortega-Guerrero, Nency P. Domingues, Miriam Jasmin Pougin, Berend Smit, Leticia Hosta-Rigau, Chris Oostenbrink

**Affiliations:** † DTU Health Tech, Center for Nanomedicine and Theranostics, Technical University of Denmark, Nils Koppels Allé, Building 423, Kgs. Lyngby 2800, Denmark; § Department of Pharmacy, Shanghai University of Medicine and Health Sciences, Zhouzhu Hwy 279, Shanghai 201318, China; ∥ Nanotech@surfaces Laboratory, Empa - Swiss Federal Laboratories for Materials Science and Technology, Dübendorf 8600, Switzerland; ‡ Laboratory of Molecular Simulation (LSMO), Institut des Sciences et Ingénierie Chimiques, Valais (ISIC), École Polytechnique Fédérale de Lausanne (EPFL), Sion 1950, Switzerland; # Institute for Molecular Modelling and Simulation, Department of Material Sciences and Process Engineering, University of Natural Resources and Life Sciences, Vienna, Vienna1190, Austria

## Abstract

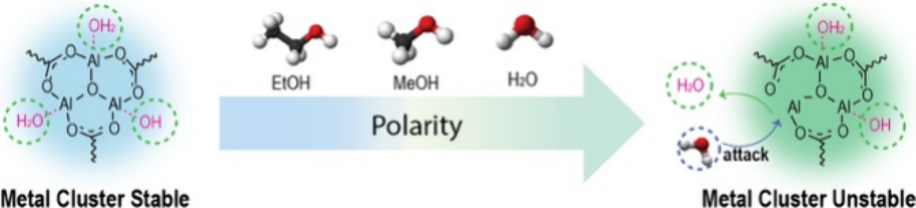

Mesoporous metal–organic frameworks (MOFs) have been recognized as powerful platforms for drug delivery, especially for biomolecules. Unfortunately, the application of MOFs is restricted due to their relatively poor stability in aqueous media, which is crucial for drug delivery applications. An exception is the porous coordination network (PCN)-series (e.g., PCN-333 and PCN-332), a series of MOFs with outstanding stability in aqueous media at the pH range from 3 to 9. In this study, we fabricate PCN-333 nanoparticles (nPCN) and investigate their stability in different solvents, including water, ethanol, and methanol. Surprisingly, the experimental characterizations in terms of X-ray diffraction, Brunauer–Emmett–Teller (BET), and scanning electron microscopy demonstrated that nPCN is not as stable in water as previously reported. Specifically, the crystalline structure of nPCN lost its typical octahedral shape and even decomposed into an irregular amorphous form when exposed to water for only 2 h, but not when ethanol and methanol were used. Meanwhile, the porosity of nPCN substantially diminished while being exposed to water, as demonstrated by the BET measurement. With the assistance of computational simulations, the mechanism behind the collapse of PCN-333 is illuminated. By molecular dynamics simulation and umbrella sampling, we elucidate that the degradation of PCN-333 occurs by hydrolysis, wherein polar solvent molecules initiate the attack and subsequent breakage of the metal–ligand reversible coordination bonds.

## Introduction

Metal–organic frameworks (MOFs), as a new class of porous materials, have attracted extensive attention in different applications, such as drug delivery, biosensing, catalysis, and gas storage and separation.^1−5^ Thanks to their high crystallinity, adjustable porosity, and tunable chemical functionality, the use of MOFs has developed rapidly over the past two decades. Many mesoporous MOF-based nanoparticles (nMOFs) are being developed for drug delivery.^6,7^ As we know, biomolecules are fragile entities sensitive to the environment and are apt to be degraded in the human body. Therefore, an appropriate delivery system is essential to ensuring their therapeutic efficacy. nMOFs can accommodate biomolecules in confined cages, thus protecting them from degradation in adverse environmental conditions, preventing dissociation due to steric restriction, and enhancing their bioactivities. Ma and co-workers pioneered enzyme incorporation within MOF-cages. They achieved this by encapsulating microperoxidase-11 (MP-11, 3.3 × 1.7 × 1.1 nm) within a Tb-based MOF with cages that have pore sizes of 3.9 and 4.7 nm and apertures of 1.3 and 1.7 nm, respectively. MOF encapsulation led to significant enhancement in both MP-11 stability and activity. This is underscored by the superior catalytic activity exhibited by encapsulated MP-11 even after 25 h of incubation compared to the free enzyme. It also demonstrated the ability of the encapsulated MP-11 to withstand six cycles of catalysis with a negligible effect on the initial reaction rate.^8^ Zhou’s group reported another interesting example by making use of porous coordination network (PCN)-888, an MOF with, so far, the largest cages (i.e., of pore sizes 6.2, 5.0, and 2.0 nm) reported.^9^ PCN-888 was used to selectively encapsulate glucose oxidase (6.0 × 5.2 × 7.7 nm) and horseradish peroxidase (4.0 × 4.4 × 6.8 nm). The encapsulated enzymes exhibited improved stability compared to free enzymes at physiological temperatures (37 °C) and in the presence of degrading trypsin.

The incorporation of biomolecules into mesoporous nMOFs is mainly conducted through postencapsulation.^10^ For that, the size of the cages and the pore aperture must at least match the size of the biomolecules of interest. Another essential parameter is the stability of nMOFs under physiological conditions to preserve well-defined cages. Surprisingly, among approximately 20,000 MOFs discovered to date, only a limited number exhibits the stability in the aqueous environment required for biomedical applications.^11,12^ Since MOFs comprise metal nodes and organic linkers, their chemical stability highly depends on the bond strength between these entities. However, the coordination bonds between metal ions and ligands are reversible, making these interactions highly vulnerable to disruptions by molecules possessing stronger coordination capabilities, such as water (H_2_O). This vulnerability can lead to deformation and even collapse of the crystalline structure. The strength of coordination bonds can usually be predicted by Pearson’s hard–soft acid–base (HSAB) theory.^13,14^ Therefore, a well-known strategy is to employ hard Lewis bases such as high valence metals (e.g., Fe^3+^ and Al^3+^ with carboxylic-based linkers) to get strong metal–ligand coordination bonds that will result in a stable MOF structure. However, even when the coordination bonds are built up with such metal ions and linkers, the stability of the MOF can also be compromised when linkers are of a certain length.

Among the reported mesoporous nMOFs that have been used for biomolecule encapsulation, the PCN-series of MOFs (e.g., PCN-333 and PCN-332) are one of the most popular classes since they display one of the highest void volumes and largest cages reported to date. In addition, PCNs have reported outstanding stability in aqueous media in the pH range from 3 to 9.^10,15,16^ We recently reported the encapsulation of hemoglobin (Hb) within PCN-333 nanoparticles (nPCN) using Al^3+^ as the metal ion. In theory, such an approach may allow to entrap individual Hb molecules (∼5 nm in size) within the large cages of the PCN-333 structure (6 × 6 × 5 nm in size).^17−19^ By doing so, the dissociation of the Hb tetramer can be prevented due to steric restriction, and the aggregation of Hb molecules can also be hindered. In our study, we demonstrated how the biological functions of Hb (i.e., the ability to reversibly bind and release oxygen) were well preserved due to the protection of nPCN.

Due to the relevance of nPCN for encapsulating large biomolecules, we further study their stability in different solvents. Specifically, H_2_O, the most essential medium for working with biomolecules, ethanol (EtOH), a mild organic solvent that is one of the friendliest solvents for biomolecules, and methanol (MeOH), which has a polarity between H_2_O and EtOH, were selected to assess the stability of nPCN. We report that the as-prepared nPCNs lose their crystalline structure while being immersed in an H_2_O environment for a period of only 2 h. The mechanism behind structural changes of nPCN is impossible to be observed through experimental characterizations, while molecular simulations serve as a useful tool for providing molecular-level insight into structure–property relationships that occur over length and time scales inaccessible to experiments.^20,21^ In recent years, molecular dynamics (MD) simulations have been employed to study the water stability of MOFs. Greathouse and Allendorf pioneered the use of classical NPT MD simulations, where a decrease in the unit cell size of IRMOF-1 was observed in a water environment.^22^ Their simulation results demonstrated that the interactions between H_2_O and IRMOF-1 were initiated either by the replacement of oxygen atoms in the ZnO_4_ tetrahedron with oxygen from water or by the formation of hydrogen bonds with hydrogen in water. Following this study, further MD simulations have been performed to explore the interactions between water and IRMOF-1, such as classical Monte Carlo simulations and density functional theory (DFT) simulations.^23,24^ However, the time scale of simulations poses a limitation for MD studies, particularly when simulating relatively stable MOFs compared with the IRMOF series. In such cases, umbrella sampling is applied to investigate decomposition mechanisms when exploring systems with high energy barriers or rare events.^25,26^ In this study, computational simulations, including umbrella sampling, were performed to offer a unique insight into the dynamic changes occurring within the PCN-333 structure. These simulations facilitated the rationalization of its stability by assessing the free energy needed to break the metal–ligand coordination bonds. All in all, our results challenge the reported intrinsic chemical stability of the PCN-series.^15,16^

## Materials and Methods

### Materials

Aluminum chloride hexahydrate (AlCl_3_·6H_2_O), *N*,*N*′-dimethylformamide (DMF), trifluoroacetic acid (TFA), acetone, ethanol (EtOH), and methanol (MeOH) were obtained from Sigma-Aldrich (Buchs, Switzerland). 4,4′,4″-*s*-Triazine-2,4,6-triyl-tribenzoicacid (H_3_TATB) was obtained from ChemScene LLC (Monmouth Junction, NJ, USA). All chemicals were used without further purification.

### Preparation and Characterization of nPCN

#### Synthesis of nPCN

A mixture of AlCl_3_·6H_2_O (18 mL, 3 mg mL^–1^ in DMF), H_3_TATB (18 mL, 1 mg mL^–1^ in DMF), and TFA (60 μL) was mixed well before being placed in an oven (Carbolite, Carbolite Ltd., United Kingdom) at 95 °C for 24 h. The heating and cooling rates were 2 and 1.5 °C min^–1^, respectively. The resulting nPCNs were then collected and washed consecutively with DMF (3×, 20 min, 15,000*g*) and acetone (soak >4 h for each washing step, 3×, 20 min, 15,000*g*) for solvent exchange and then dried in a vacuum oven (VACUTHERM, Thermo Scientific, Germany) at 60 °C for further use.

#### Stability Study of nPCN in Different Solvents

First, the dried nPCN powders were resuspended in acetone assisted with mild stirring for 2 h at room temperature until homogeneous nPCN-suspensions were obtained. For solvent replacement, the suspension was split into three batches and washed in EtOH, MeOH, or H_2_O (3× , 20 min, 15,000*g*). Then, the nPCN-suspensions were soaked in different solvents for predesigned time intervals, i.e., 1 and 7 days at room temperature. Various EtOH:H_2_O mixtures at volume ratios of 75:25, 50:50, and 25:75 were also considered (i.e., 75EtOH, 50EtOH, and 25EtOH, respectively) and tested for a duration of 1 day. After the treatment, the solvents of nPCN-suspensions were replaced with acetone (2× , 20 min, 15,000*g*) again and dried under vacuum at 60 °C for further characterization.

#### Characterization of nPCN

The morphology of the obtained nPCNs was observed by using scanning electron microscopy (SEM, Thermo Fisher Scientific Teneo, Massachusetts, USA) at an accelerating voltage of 3 kV and a beam current of 13 pA.

The crystalline structure of nPCN after being soaked in different solvents was measured using an X-ray diffraction (XRD) instrument (Bruker D8 Advance, Bruker, Germany). The measurements were performed at room temperature (40 kV, 40 mA, 0.02°/2θ step) using monochromated Cu Kα (λ = 1.5418 Å) radiation. The simulated XRD pattern of PCN-333(Al) was generated from the corresponding crystallographic information file (CIF) (Cambridge Crystallographic Data Centre (CCDC) code 997911) by using Mercury 3.0.

The porosity of nPCN was detected using a High Precision Surface Area and Pore Size Analyzer BELSORP-Mini II (Microtrac BEL, Japan), and the data were analyzed using Brunauer–Emmett–Teller (BET) analysis models. Specifically, the dried nPCN powders (approximately 15 mg per sample) were activated at 200 °C for 12 h under a dynamic vacuum; subsequently, nitrogen (N_2_) adsorption/desorption isotherms were collected at 77 K. The obtained adsorption isotherms were analyzed using the BELMaster software (version 7.2.0.4) with the Barrett, Joyner, and Halenda (BJH) model to determine the surface area and pore size distribution.

### Computational Methods

#### Model Construction

The crystal structure of PCN-333(Al) was taken from the CCDC with deposition code 997911. First, the obtained structure was cleaned by removing all the solvent residues. Next, PCN-333(Al) was cut into the smallest unit (triclinic box, dimension *a*/*b*/*c* = 89.4 nm, angle α/β/γ = 60°) that contains the characteristics of the crystalline structure. To neutralize the MOF system, one OH^–^ and two H_2_O were added to each metal node to fill the open metal sites.

The geometric properties of the framework model were evaluated using the software Zeo++.^27^ The pore volume was accessed using the probe-occupiable pore volume model, as implemented in the software. This technique was developed by Ongari et al.,^28^ providing a computational pore volume definition directly related to experimental pore volumes obtained from N_2_ isotherms.

#### Interaction Function Force Field

Force field parameters for the linker H_3_TATB were generated using the bb_editor tool of GROMOS^29^ and were assigned according to the 54A8 parameter set of the GROMOS force field.^30^ Bonded parameters, atom types, and initial partial charges for the linker were assigned by analogy to similar functional groups in the force field. A short, 2 ns, initial simulation of the PCN structure in H_2_O was performed using the formal charges of +3 for Al and −2 for the central oxygen. From the last 200 ps of this simulation, 20 configurations were used to extract a total of 1356 reduced models of the Al nodes. These reduced systems contained any atoms within 0.5 nm of the central Al_3_O cluster of the node, including additional solvent molecules. Dangling bonds were saturated with hydrogen atoms. The 1356 node structures were submitted to single-point density functional calculations in Gaussian16^31^ within the unrestricted Kohn–Sham formalism using the PBE functional^32^ with OPTX exchange^33^ and the def2-SV(P) basis set.^34^ Partial charges were estimated using the Merz–Singh–Kollman scheme,^35^ using UFF radii and averaged over the 1356 models and over the chemical symmetry of the nodes. The resulting charge distributions amounted to a charge of −0.956 *e* on the central O, +1.435 *e* on the three Al groups, and −0.3915 *e* on the six coordinating carboxylate groups, leading to a net charge of +1 for the node. The metal chelating interaction of Al–O bonds is subsequently described solely by the balance of electrostatic and Lennard-Jones interactions. Solvents were modeled according to the SPC model for H_2_O,^36^ ethanol, and methanol as derived for the GROMOS force fields.^37^ Complete topologies are available, as outlined in the data availability section.

#### Molecular Dynamics (MD) Simulation

All the MD simulations were performed using the GROMACS simulation software (version 2022.1).^38^ The PCN-box was filled with the solvents EtOH, MeOH, and H_2_O. For validation of the force field for PCN-box simulations, the PCN-333(Al) unit was simulated in EtOH, MeOH, and H_2_O under a constant volume (NVT) ensemble at 300 K for 100 ps followed by a 10 ns MD simulation. Short-ranged nonbonded interactions were cut off at 1.4 nm. The temperature was kept constant at 300 K using v-rescale temperature coupling. The bond lengths were fixed using the LINCS algorithm, and long-range electrostatics were calculated using the particle mesh Ewald (PME) algorithm. The atom-positional root-mean-square deviation (RMSD) of the simulated structure compared to the starting reference structure was computed over the simulation. Detailed information about the simulation settings (.mdp files) is available as outlined in the data availability section.

#### MD Simulations with Distance Restraints

As described above, the PCN-box was filled with the solvents EtOH, MeOH, and H_2_O. The distances between an oxo-oxygen atom and a coordinated metal atom (i.e., Al) were defined as 0.3 and 0.5 nm, respectively, and a distance restraint simulation was conducted in Gromacs. This was followed by a normal NVT simulation in which the distance restraints were removed. The trajectory of the target atoms was reviewed using the visual molecular dynamics (VMD) tool.

#### Umbrella Sampling Simulation

The PCN-box was filled with the solvents EtOH, MeOH and H_2_O. Following the steepest descent minimization, the system was equilibrated for 100 ps under an NVT ensemble at 300 K. The structure obtained from NVT equilibration was used as the starting configuration for the pulling simulation. A metal atom (i.e., Al), located in the center of the (periodic) MOF-box, was selected. The target metal atom was defined as Group 1, while the remaining atoms of the metal node were defined as Group 2 (M2(μ3-O)(COO)6). The Group 1 atom (target metal atom) was pulled away from the core structure of Group 2 along the *y* axis. The corresponding oxo-oxygen atom was used as the reference atom for the treatment of the distance restraint using a spring constant of 30,000 kJ mol^–1^ nm^–2^, a pull rate of 0.1 nm ps^–1^ at a time step of 0.002 ps for integration over 4 ps. The starting distance between the target Al-atom (Group 1) and the oxo-oxygen atom (within Group 2) was approximately 0.2 nm, and a distance of approximately 0.6 nm was achieved after the pulling operation (the settings for the pulling process are available, as outlined in the data availability section). Snapshots were taken from the trajectories during the pulling process to generate the starting configurations for the umbrella sampling windows. A window space of 0.025 nm was applied, and about 17 windows were used for further umbrella sampling simulation. At each window, 100 ns of NVT MD was performed (the settings for the umbrella sampling process are available as outlined in the data availability section) and then a weighted histogram analysis method (WHAM) was applied to compute the potential of mean force (PMF).^39^

## Results and Discussion

### Synthesis and Characterization of PCN-333(Al) Nanoparticles (nPCN)

**Figure 1:**
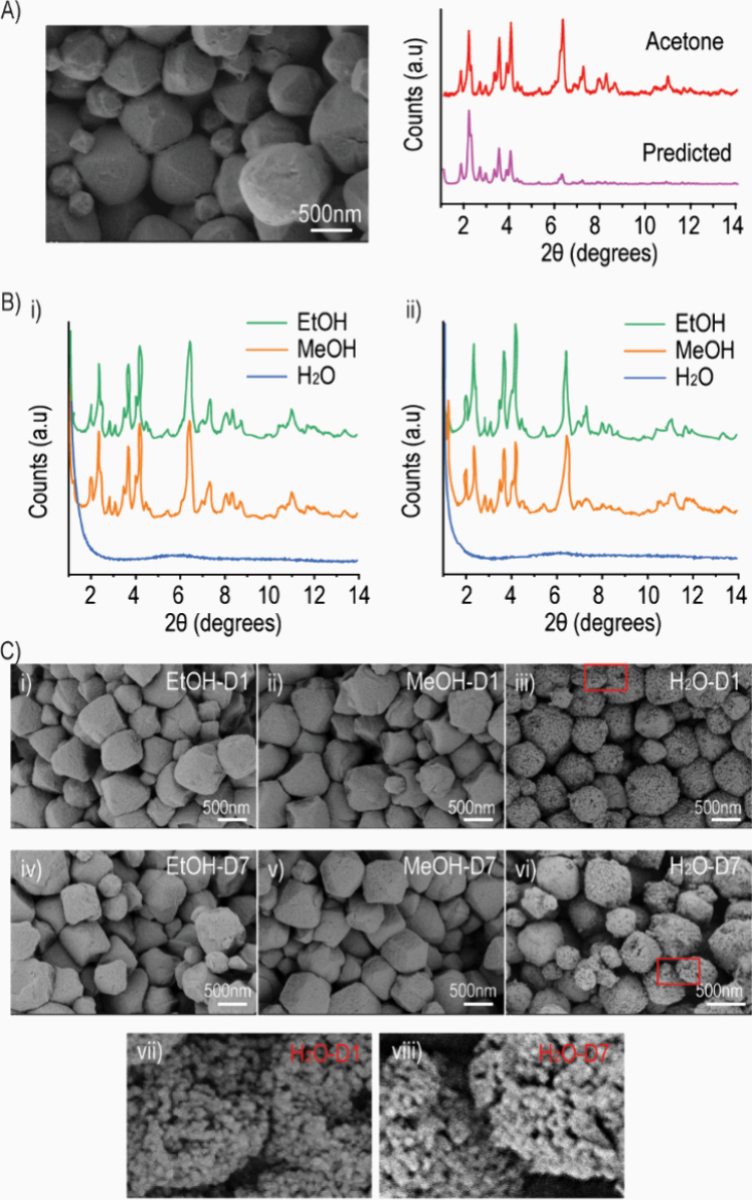
Characterization of nanosized-PCN-333(Al) (nPCN) in different solvents. (A) SEM images of nPCN and the XRD patterns as prepared and as predicted. (B) XRD patterns of nPCN after soaking in different solvents for 1 day (D1) (i) and 7 days (D7) (ii). (C) SEM images of nPCN after soaking in different solvents for D1 and D7, and the enlarged images of nPCN after soaking in water (H_2_O) for D1 (vii) and D7 (viii).

PCN-333(Al), which is constituted by trivalent metal species (i.e., Al^3+^) and the organic linker H_3_TATB, features one of the highest void volumes and largest cages among the mesoporous MOF materials reported to date.^15,40^ These features make nPCN one of the most widely used mesoporous platforms for biomolecule encapsulation.^16,41^ The well-regulated cages formed owing to their crystalline structure enable the encapsulation of biomolecules by a postencapsulation method. In this study, the freshly synthesized nPCN displayed a characteristic octahedral shape as previously reported^15^ and the crystalline structure of synthesized nPCN was evidenced by the XRD results since its XRD pattern was identical to the one predicted from the CIF of PCN-333(Al) from CCDC (Figure 1A).

### Stability of nPCN in Different Solvents

The stability of the crystalline structure of nMOFs is very important for biomedical applications since it is a prerequisite to guarantee the successful accommodation of biomolecules into well-organized cages by postencapsulation. Given the fragility and sensitivity of biomolecules to organic solvents, it is imperative that nMOF exhibits stability not only in physiological conditions but also particularly in aqueous media, which serve as the essential working environment for biomolecules. Therefore, the preference lies in utilizing aqueous media and mild organic solvents when working with biomolecules to maintain their conformational integrity. As shown in Figure 1B, for the solvents of EtOH and MeOH, the XRD patterns matched well with the predicted XRD calculated from the CIF of PCN-333(Al). This indicates that the crystalline structure of nPCN was preserved after being soaked in EtOH and MeOH for both 1 and 7 days. Interestingly, the effect of H_2_O on the nPCN crystalline structure was remarkably different, as shown by the XRD patterns, where the crystalline peaks were completely lost with the appearance of the typical amorphous halo pattern. A more precise XRD change of nPCN upon dispersion in H_2_O over time (i.e., 2 and 4 h) is shown in Figure S1 (Supporting Information). To validate our findings, we followed the protocol as previously reported^16^ and synthesized both PCN-333(Al) and PCN-333(Fe) particles; both of them lost their XRD patterns after soaking in H_2_O for 4 h (Figure S2 in the Supporting Information). We can conclude that the crystalline structure of nPCN collapsed rapidly in H_2_O for only 2 h. To differentiate the effects of EtOH and MeOH, an accelerated study was conducted by soaking nPCN in each solvent separately at 60 °C over 5 days. The findings presented in Figure S3 (Supporting Information) demonstrate that the structure of nPCN was more stable upon exposure to EtOH compared to MeOH. The morphology of nPCN after being soaked in the different solvents was further characterized by SEM (Figure 1C). Using EtOH as the solvent, the nPCN displayed its characteristic octahedral shape. Meanwhile, in MeOH, the morphology of nPCN also kept its octahedral shape, and small aperture cracks can be observed, especially after 7 days. The morphology of nPCN led to considerable changes when dispersed in H_2_O, as shown in Figure 1C (iii and iv). Although the overall octahedral shape was still present, it was damaged in this case, specifically degrading into small spherical segments (Figure 1C, vii and viii). It was speculated that the nPCN went through decomposition into numerous small spherical segments upon being suspended in H_2_O, and its well-organized structure was destroyed, as evidenced by the loss of crystallinity and the presence of the amorphous structure as shown by XRD. It could be concluded that nPCN was unstable in H_2_O and more stable in MeOH and EtOH.

**Figure 2:**
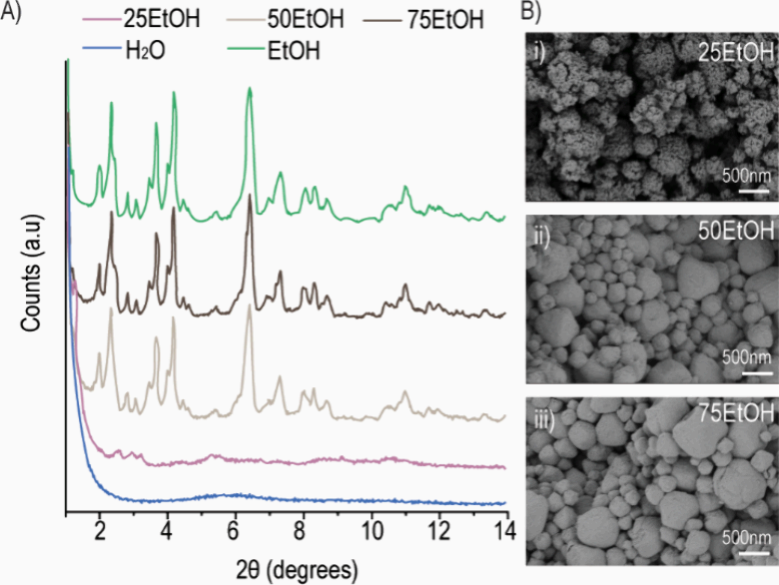
Characterization of nanosized-PCN-333(Al) (nPCN) in the mixture of EtOH and H_2_O for 1 day. (A) XRD patterns of nPCN after soaking in a solvent mixture of EtOH and H_2_O at different ratios of 25:75, 50:50, and 75:25, i.e., 25EtOH, 50EtOH, and 75EtOH, respectively. (B) SEM images of the corresponding nPCN in different solvents.

Given the results in Figure 1, to assess if the addition of EtOH was able to increase the stability of nPCN soaked in H_2_O, several EtOH:H_2_O ratios were next evaluated. At a low EtOH ratio (i.e., 25EtOH), the nPCN still displayed an amorphous pattern, where the characteristic XRD peaks were absent (Figure 2A). The decomposition of nPCN was further demonstrated by SEM images, where the intact octahedral shape disintegrated into small spherical segments with defects (Figure 2B, i). With the increase in the amount of EtOH in the solvent mixture, the crystalline structure of nPCN was preserved when the EtOH volume ratio was up to 50%, and an intact octahedral shape was observed in the corresponding SEM images (Figure 2B, ii and iii).

**Figure 3:**
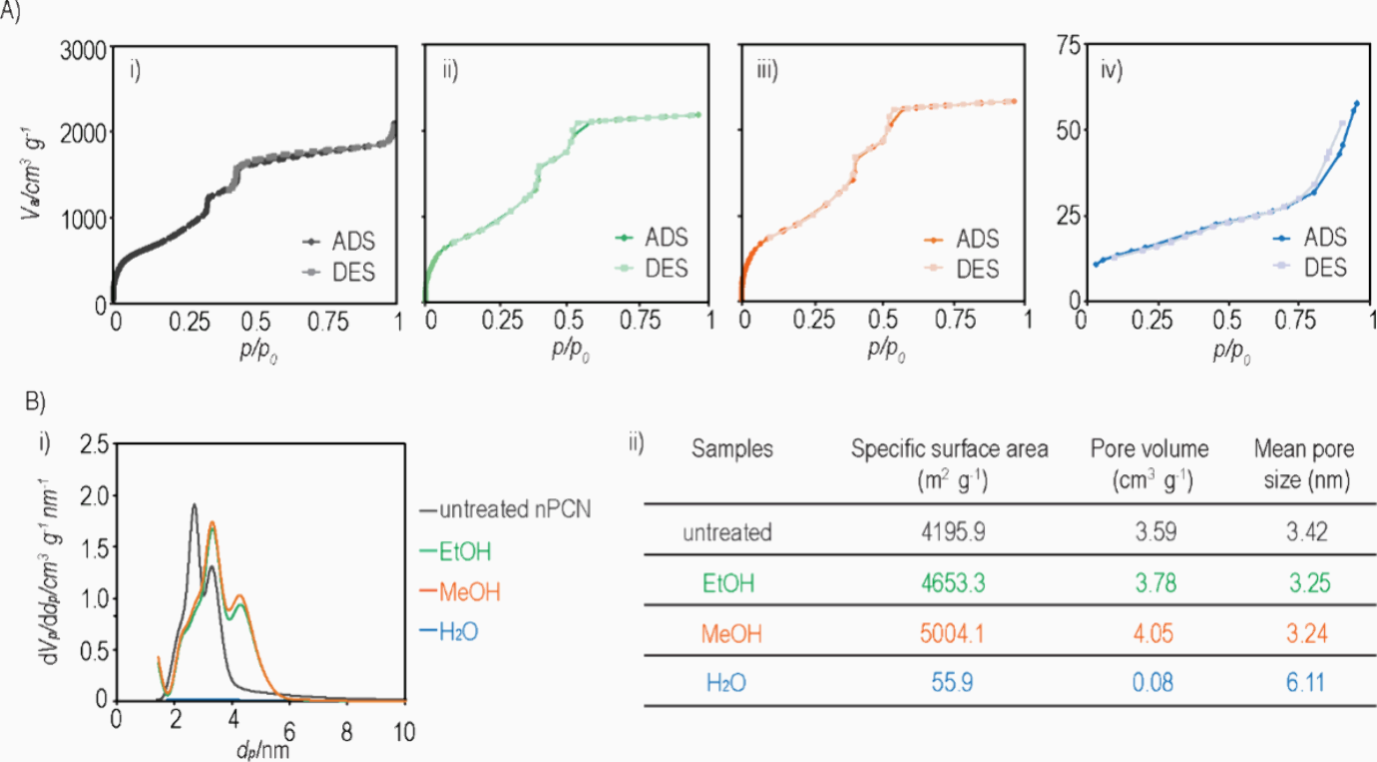
Porosity characterization of nanosized-PCN-333(Al) (nPCN) in different solvents. (A) N_2_ sorption isotherm of untreated nPCN (i) and after soaking in different solvents in EtOH (ii), MeOH (iii), and H_2_O (iv). (B) Pore size distribution (i) and porosity information (ii) of nPCN before and after soaking in different solvents.

The surface area and volume of interconnected pores are critical characteristics for drug delivery, which are determined by the well-organized crystalline structure of nPCN. To assess the porosity of nPCN, we performed N_2_ sorption at 77 K. Figure 3A (i–iii) illustrates a typical type IV isotherm associated with capillary condensation occurring in mesopore (>2 nm in diameter) materials. In contrast, a classical type II isotherm was presented for H_2_O-soaked nPCN, which was interpreted as a typical pattern for nonporous materials. The corresponding BJH surface areas were 4195.9, 4653.3, 5004.1, and 55.9 m^2^ g^–1^ for untreated, EtOH-soaked, MeOH-soaked and H_2_O-soaked nPCN, respectively (Figure 3B). The two steep increases at *p*/*p*0 = 0.4 and 0.5 (or *p*/*p*0 = 0.3 and 0.4 for untreated nPCN) on the N_2_ adsorption isotherm corresponded to the two types of mesoporous cages in PCN-333(Al). The experimental void volume of untreated nPCN was 3.59 cm^3^ g^–1^, while the value calculated from our structural model was 3.71 cm^3^ g^–1^. This calculated value was derived from a molecular simulation of a perfect crystalline structure; however, the inevitable defects in the crystallinity of the synthesized samples contribute to the observed relatively lower pore volume. Interestingly, the pore volume of nPCN increased in both EtOH and MeOH, while it dramatically diminished in H_2_O, with values of 3.78, 4.05, and 0.08 m^3^ g^–1^, respectively. For EtOH and MeOH, the increase in pore volume and surface area can be attributed to changes in the crystalline structure of PCN, such as the formation of additional pores or the enlargement of existing pores due to deformation and even partial collapse of the original structure. In contrast, the dramatic decrease in pore volume and surface area of nPCN in H_2_O is likely due to the complete collapse of its crystalline structure.

### MD Simulations of nPCN in Different Solvents

**Figure 4:**
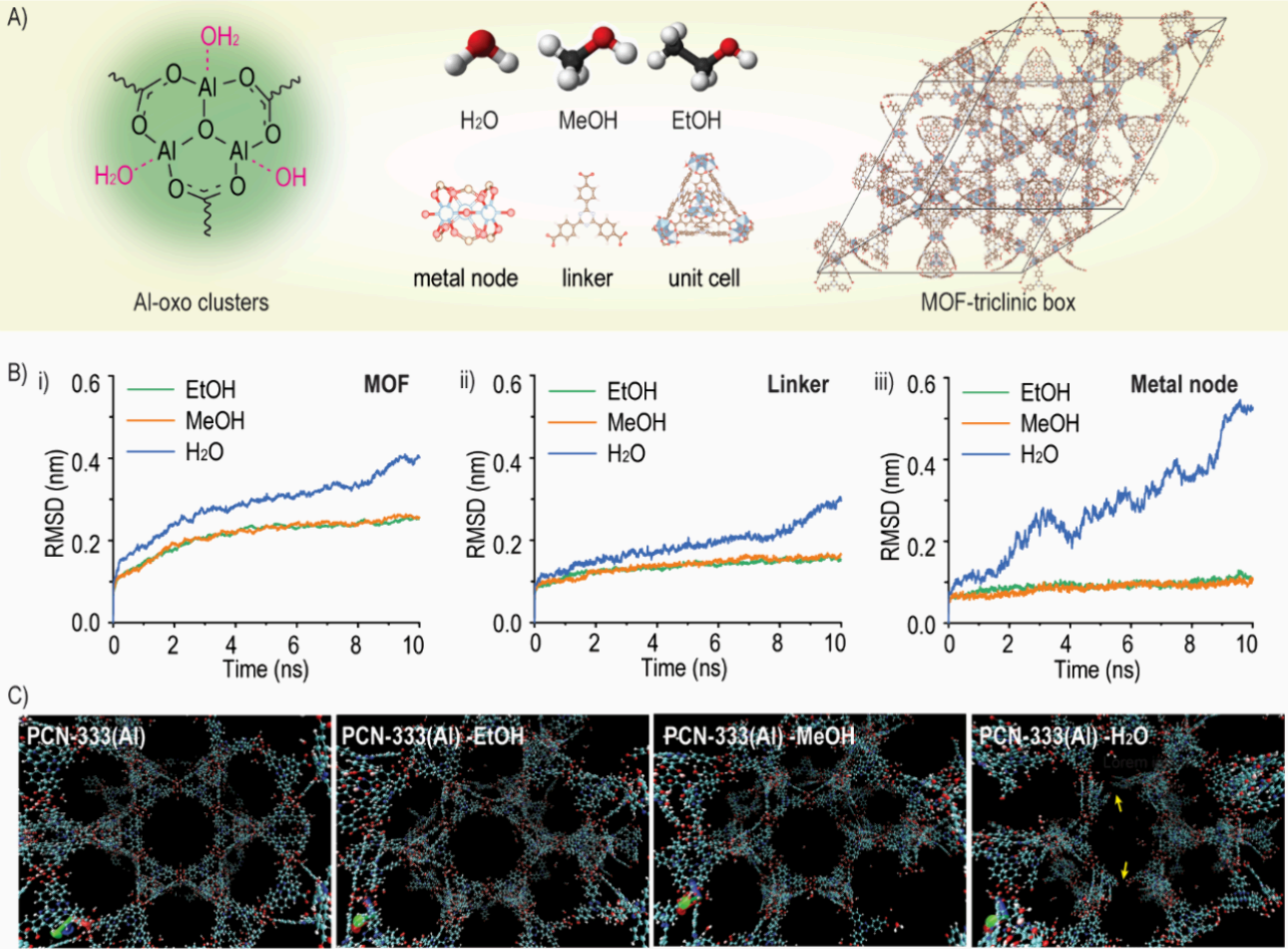
Molecular dynamics simulations of the MOF system filled with different solvents. (A) Scheme of the Al-oxo clusters, solvents, and MOF-triclinic box. (B) RMSD of the NVT simulation of the MOF structure in different solvents, including the whole simulated system of the MOF (i), linker (i.e., H_3_TATB) (ii), and metal node (iii). (C) Images of the original framework and those after 10 ns NVT simulation in different solvents, i.e., EtOH, MeOH, and H_2_O. The yellow arrows point to the broken part of the well-organized structure.

The dynamic stability of nPCN in different solvents was further explored with computational simulation using GROMACS. The structures of the units constructing the MOF crystalline structure and the MOF-triclinic box used for MD simulation are shown in Figure 4A. The RMSD values for the PCN-333(Al) backbone from its starting to the final position were calculated from the entire MD trajectory as a function of time; see Figure 4B. The RMSD values were analyzed in terms of the whole PCN system, the metal nodes (i.e., Al-oxo-clusters), and the linkers (i.e., H_3_TATB).

The PCN-333(Al) structure reached equilibrium quickly and then experienced turbulence during the following NVT simulation in H_2_O. An overall comparison of the RMSD values showed that the MOF structure was much more stable in EtOH and MeOH during the entire simulation compared to the structure filled with H_2_O. As compared to H_2_O, relatively low RMSD values of 0.25, 0.15, and 0.11 nm were displayed for the whole MOF system, linkers, and metal nodes for both EtOH and MeOH, respectively. For the MOF in H_2_O, the RMSD values were 0.40, 0.30, and 0.52 nm for the whole MOF system, linkers, and metal nodes, respectively, at the end of the simulation with a clear continuous drift. It can be assumed that the decomposition of the MOF structure was apt to occur from the metal node sites since the distance of paired metal atoms rose rapidly during the simulation with an RMSD value of up to 0.52 in 10 ns. For the linker and whole MOF system, following a slow continuous ascent, a sharp rise was observed at 8 ns, with the explanation that the whole MOF crystalline structure might start to collapse. The corresponding structures after NVT simulation in different solvents are shown in Figure 4C, where a well-organized crystalline structure was observed for EtOH, a slight deformation of the framework was observed for MeOH, and an obviously broken framework was present for H_2_O.

### MD Simulations of nPCN with Distance Restraints in Different Solvents

**Figure 5:**
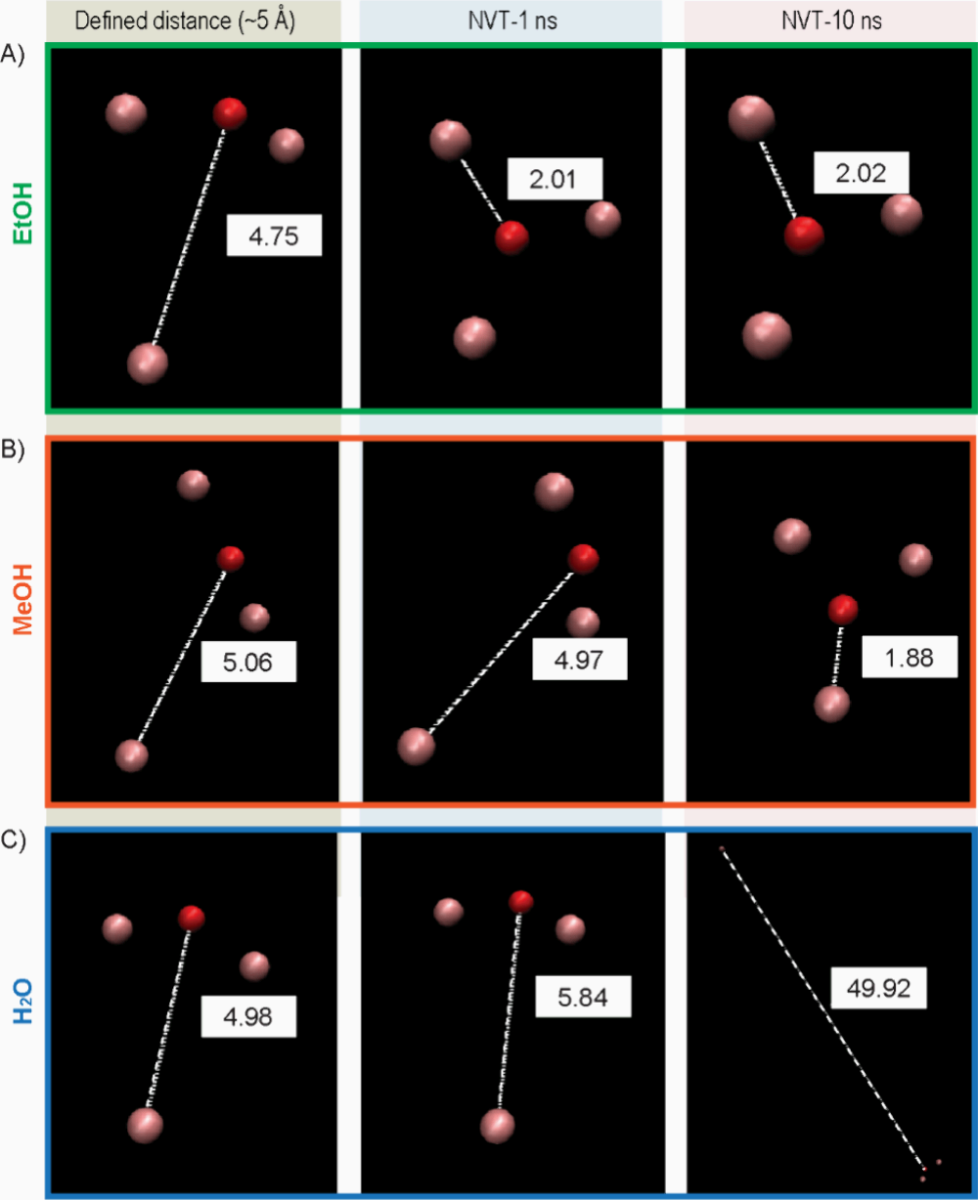
Movement of atoms that are composed of the metal node while enforcing a distance of oxo-oxygen (O) and aluminum (Al) of approximately 5 Å followed by NVT simulations after removing the distance restraint for 1 and 10 ns. The simulation box is filled with (A) EtOH, (B) MeOH, and (C) H_2_O. The pink spheres are Al atoms, and the red sphere is the O atom.

The original distance between the oxo-oxygen atom and its coordinated metal atom (i.e., Al) is approximately 0.2 nm. To disrupt the structure, we set restraint distances of the atoms of interest to 0.3 and 0.5 nm. When the restraint distance was set to 0.3 nm, all atoms returned to their original positions after removal of the distance restraint during a 1 ns NVT simulation (data not shown). Interestingly, when the distance was increased to 0.5 nm, all the atoms returned to their original positions in the EtOH environment within 1 ns, as shown in Figure 5A. In the MeOH environment, this return occurred within 10 ns. However, in the H_2_O environment, the metal atom “flew away” from the metal node, behaving as a free atom. This phenomenon indicates that the attraction interactions between the oxo-oxygen atom and the coordinated metal atom are influenced by the solvent environment.

### Umbrella Sampling Simulations of nPCN in Different Solvents

**Figure 6:**
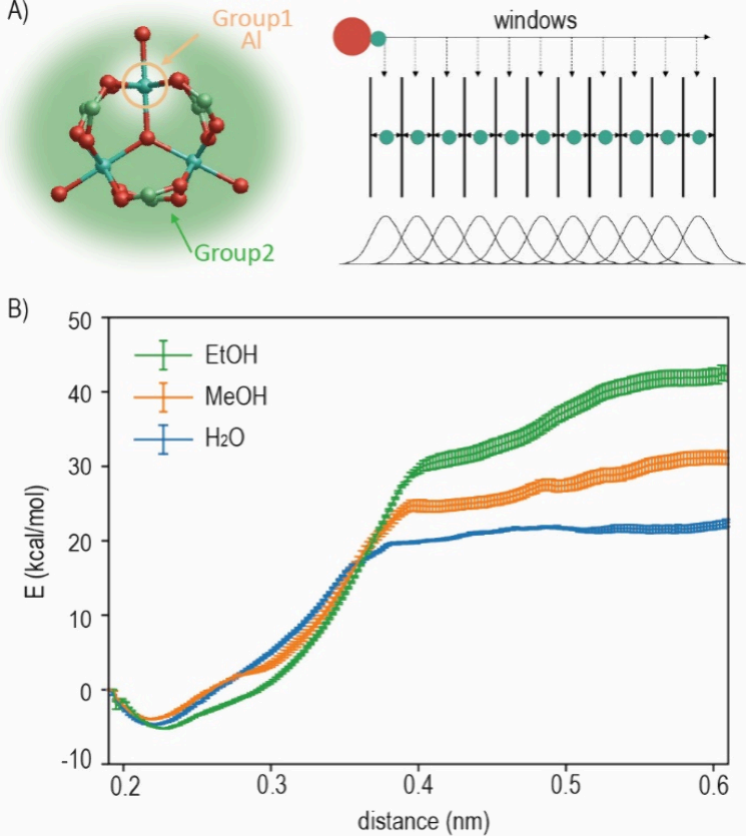
Umbrella sampling simulations of the MOF system filled with different solvents. (A) Scheme of the defined Group 1 and Group 2 for MD simulations and the mechanism of umbrella sampling simulations. (B) Potential of mean force (PMF) of PCN-333(Al) in terms of pulling one chelating metal atom (i.e., Al) away from the metal node in the presence of different solvents.

To gain a deeper understanding of the stability of PCN-333(Al) in different solvents, the free energy cost of moving a target Al atom away from its initial structure was determined. This analysis was accomplished by extracting the potential of mean force (PMF) from a series of umbrella sampling simulations. The output data recorded the movement of the target metal ion away from its coordinated metal node, the histograms of which were analyzed by the weighted histogram analysis method (WHAM), see Figure S4 (Supporting Information). Since all of the individual histograms were smooth and showed partial overlap, the selected umbrella sampling windows were considered adequate for the PMF calculation. The PMF extracted by the WHAM is shown in Figure 6. The minimum energy state of the PCN-333(Al) occurred at a distance of approximately 0.22 nm, which is in good agreement with the van der Waals radius between nonbonded components. The pulling process has to overcome the attractive force between the coordination of Al-oxo, and consequently, the PMF of the MOF system increases during the pulling process. The target Al atom was pulled away up to 0.6 nm, and energy barriers of 47.3, 34.8, and 27.2 kcal mol^–1^ were obtained for the system filled with solvent EtOH, MeOH, and H_2_O, respectively.

According to the Eyring equation, reactions with an activation barrier around empirical 20 kcal mol^–1^ take about 1–2 min. With an activation barrier over 25 kcal mol^–1^, it takes days or weeks to complete. Therefore, it can be concluded that PCN-333(Al) was generally a stable crystalline material in aqueous media. However, upon immersion in H_2_O, there is a high possibility that the crystalline structure decomposes spontaneously and that a decrease in the structure is observed over time. Nevertheless, while using EtOH and MeOH, PCN-333(Al) stability was strongly enhanced, and the most stable PCN-333(Al) was obtained when EtOH was used as the solvent, which was in line with the experimental observations.

### MOF Stability

Since their first synthesis in 2015 by Zhou’s group, the PCN-series of MOFs has been widely investigated as an excellent platform for biomolecular encapsulation. In our group, we have used protein-loaded nPCN as functional therapeutic nanocarriers. However, the data presented in this study reveal changes in morphology, a loss of the characteristic XRD patterns, and a porosity change of nPCN upon exposure to H_2_O. These results raise important questions regarding the stability of nPCN in different solvents.

Generally, exposure to H_2_O can induce the collapse of MOFs through processes such as hydrolysis and capillary-driven force, as reported in previous studies.^42,43^ Hydrolysis occurs when H_2_O molecules attack the reversible coordination bonds between the linkers and metal nodes. Another destructive process arises from capillary forces where solvent molecules with high surface tension can adhere strongly to the MOF’s cavities or channels via hydrogen bonds, resulting in channel collapse during the activation process. To mitigate the influence of capillary-driven forces, we employed a solvent exchange strategy, initially dispersing the dried nPCN powders in the low-polarity solvent acetone before exchanging into the solvents of interest for treatment at certain time intervals. Following this, we replaced the solvents with acetone once more before vacuum-drying. By doing this, we exclude the destruction induced by solvent capillary force during the activation process. Therefore, the capillary-driven force was not the dominant factor contributing to the collapse of nPCN in this study.

Next, the instability pathway of nPCN is further investigated with the assistance of computational simulations. MD simulations allow us to explore nPCN’s behavior independent of capillary force, which is impossible to achieve through conventional experimental approaches. The simulations provide valuable insights into the interactions between solvent molecules and MOF crystalline structures. The RMSD results from the MD simulation demonstrated that the collapse of the crystalline structure occurred in the metal node region when exposed to H_2_O, but not in EtOH and MeOH. Further analysis using distance restraint simulation revealed strong interactions between the oxo-oxygen atom and the coordinated metal atom in EtOH (returning to the original positions in 1 ns), weaker interactions in MeOH (returning in 10 ns), and a complete loss of attraction in H_2_O. This suggests that hydrolysis occurs as H_2_O molecules disrupt the metal–ligand coordination bonds, competing effectively with the oxo-oxygen interactions. To evaluate the stability of nPCN’s crystalline structure in different solvents, umbrella sampling was employed. The PMF analysis revealed that the energies required to break the metal–ligand coordination bond were 47.3, 34.8, and 27.2 kcal mol^–1^ for systems filled with EtOH, MeOH, and H_2_O, respectively. These energy barriers correlate with our empirical observations that hydrolysis is less likely in EtOH and MeOH, but more readily occurs in pure H_2_O. It is important to note that one key consideration of this kind of periodic boundary condition used in MD simulation is that they only account for an infinite, perfect crystal structure, thereby neglecting experimental properties such as internal defects and external surface area.

Overall, we can conclude that the stability of nPCN when exposed to different solvents follows the order of EtOH > MeOH > H_2_O based on both experimental characterization and MD simulations.

## Conclusions

In this study, a combination of experimental characterizations and computational simulations was performed to investigate the stability of nPCN in different solvents. The elucidation by experimental characterization in terms of XRD, SEM, and BET confirmed the high void volume and large cages of the as-prepared nPCN. The stability of nPCN when exposed to different solvents follows the order EtOH > MeOH > H_2_O, based on both experimental characterization and MD simulations. Free-energy calculations serve as useful tools for obtaining insights into the free-energy changes associated with conformational transitions, thereby providing valuable stability information for the structures of interest. To the best of our knowledge, the application of PMF calculations in evaluating the stability of crystalline MOF structures has not yet been investigated. This presents a promising avenue for further exploration in future research.

## Data Availability

The data underlying this study are openly available via Zenodo at 10.5281/zenodo.10970489, which includes the relevant topologies and input files.

## Supporting Information Available

Figures showing the change of the XRD patterns of nPCN-333 in water over time, the change of XRD patterns of PCN-333(Al) and PCN-333(Fe) following the protocol as previously reported, the change of the XRD pattern of nPCN in EtOH and MeOH at 60 °C, and the histograms for each umbrella sampling simulation (PDF)
