# Supplementary material for: Stability Assessment in Aqueous and Organic Solvents of Metal–Organic Framework PCN 333 Nanoparticles through a Combination of Physicochemical Characterization and Computational Simulations
Source: Langmuir. 2024 Oct 12;40(42):21976–84. doi: 10.1021/acs.langmuir.4c01684 (PMC11500399; doi:10.1021/acs.langmuir.4c01684)
Supplement: Supplementary file 1 — la4c01684_si_001.pdf [file la4c01684_si_001.pdf]

Supporting Information

Stability assessment in aqueous and organic solvents of metal-organic framework PCN 333 nanoparticles through a combination of physicochemical characterization and computational simulations

Xiaoli Liu[†§], Andres Ortega-Guerrero[||], Nency P. Domingues[‡], Miriam Jasmin Pougin[‡], Berend Smit[‡], Leticia Hosta-Rigau[†], Chris Oostenbrink[#*]

† DTU Health Tech, Center for Nanomedicine and Theranostics, Technical University of Denmark, Nils Koppels Allé, Building423, Kgs. Lyngby 2800, Denmark

§ Department of Pharmacy, Shanghai University of Medicine and Health Sciences, Zhouzhu Hwy 279, Shanghai 201318, China

|| Nanotech@surfaces Laboratory, Empa - Swiss Federal Laboratories for Materials Science and Technology, Dübendorf 8600, Switzerland

‡ Laboratory of Molecular Simulation (LSMO), Institut des Sciences et Ingénierie Chimiques, Valais (ISIC), École Polytechnique Fédérale de Lausanne (EPFL), Sion 1950, Switzerland

Institute for Molecular Modeling and Simulation, Department of Material Sciences and Process Engineering, University of Natural Resources and Life Sciences, Vienna, Vienna 1190, Austria

**Email: chris.oostenbrink@boku.ac.at*

KEYWORDS

metal-organic frameworks (MOF), stability, aqueous environment, molecular dynamics simulation, umbrella sampling

Method

Preparation of PCN-333(Al) and PCN-333 (Fe):

As previously reported,[1] 10 mL DMF solution of $AlCl_3 \cdot 6H_2O$ (1.5 mg mL^{-1}), 5 mL DMF solution of H_3TATB (1 mg mL^{-1}), 15 mL DMF and 50 μL TFA was mixed and heated at 95 °C for 24 h. nPCN-333 (Al) was collected by centrifugation.

10 mL DMF solution of $FeCl_3$ (1.5 mg mL^{-1}), 5 mL DMF solution of H_3TATB (1 mg mL^{-1}), 15 mL DMF and 50 μL TFA was mixed and heated at 120 °C for 24 h. nPCN-333 (Fe) was collected by centrifugation.

Result

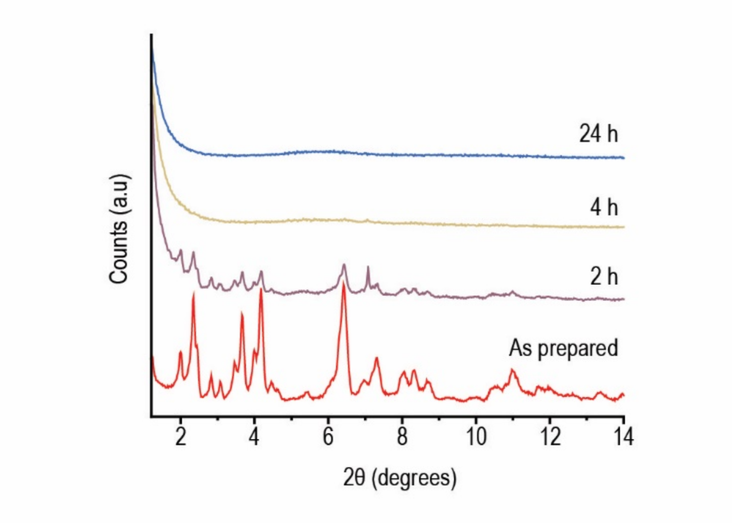

Figure S1 The change of the XRD patterns of nanosized-PCN-333 (Al) while soaking in water over time.

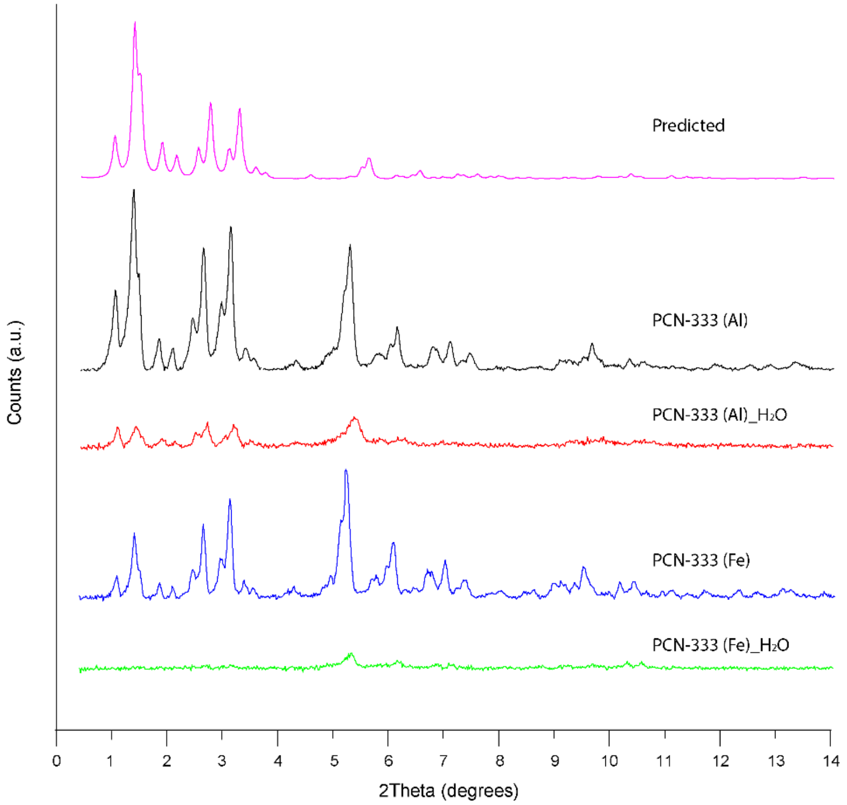

Figure S2 The XRD patterns of PCN-333(Al) and PCN-333 (Fe) particles before and after soaking in H_2O for 4h.

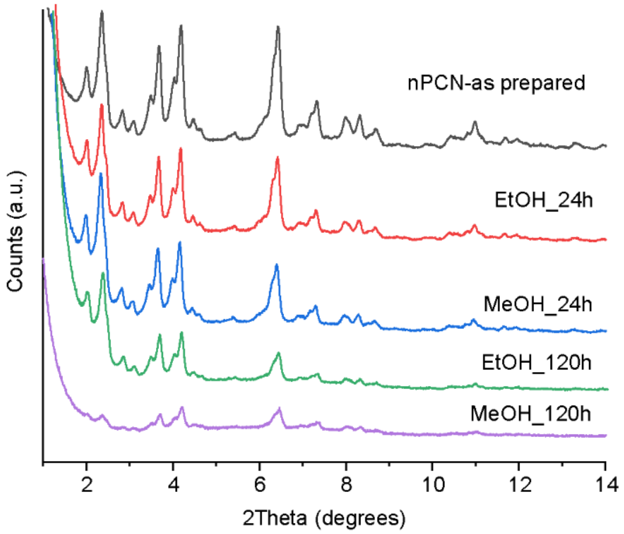

Figure S3 The XRD patterns of nPCN soaking in EtOH and MeOH at 60 °C for 24h and 120h.

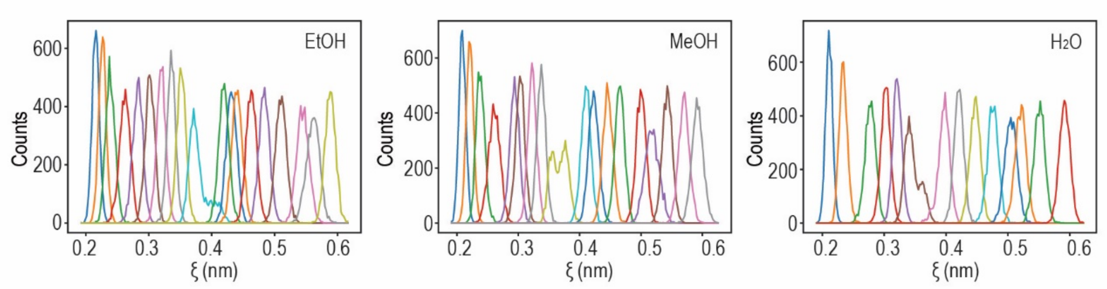

Figure S4 The histograms for each umbrella sampling simulation.

Reference

(1) Lian, X.; Erazo-Oliveras, A.; Pellois, J. P.; Zhou, H. C. High Efficiency and Long-Term Intracellular Activity of an Enzymatic Nanofactory Based on Metal-Organic Frameworks. *Nat Commun* 2017, *8* (1) 6969-6973. https://doi.org/10.1038/s41467-017-02103-0.
